# Supplementary material for: Tackling Food Insecurity in Cabo Verde Islands: The Nutritional, Agricultural and Environmental Values of the Legume Species
Source: Foods. 2021 Jan 20;10(2):206. doi: 10.3390/foods10020206 (PMC7909421; doi:10.3390/foods10020206)
Supplement: Supplementary file 1 [file foods-10-00206-s001.pdf]

Supplementary Materials:

Figure S1: Santiago Island and details on market locations (left). List of the 15 Cabo Verde bean accessions used for this study, including local names in Cabo Verdean Creole; origin/market location and altitude in Santiago Island (right).

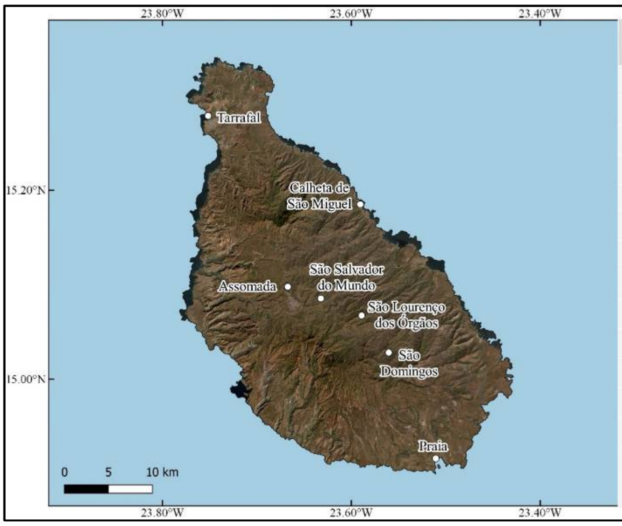

Accession	Species	Local name (Cabo Verde Creole)	Origin (in Santiago Island)	Altitude (m)
CV01Cc	*Cajanus cajan*	Feijão congo	Assomada	560
CV02Cc	*Cajanus cajan*	Feijão congo	Assomada	560
CV01Lp	*Lablab purpureus*	Feijão pedra	Assomada	560
CV02Lp	*Lablab purpureus*	Feijão pedra	Tarrafal	25
CV03Lp	*Lablab purpureus*	Feijão pedra	Calheta	10
CV04Lp	*Lablab purpureus*	Feijão pedra	Praia	30
CV05Lp	*Lablab purpureus*	Feijão pedra	São Jorge dos Orgãos	290
CV06Lp	*Lablab purpureus*	Feijão pedra; feijão creca	São Salvador do Mundo	440
CV01Pl	*Phaseolus lunatus*	Feijão bonge; imbonge fava	Assomada	560
CV02Pl	*Phaseolus lunatus*	Feijão bonge; imbonginho	São Salvador do Mundo	440
CV01Pv	*Phaseolus vulgaris*	Sapatinha	Assomada	560
CV02Pv	*Phaseolus vulgaris*	Sapatinha	Assomada	560
CV01Vu	*Vigna unguiculata*	Feijão bongolon	Assomada	560
CV02Vu	*Vigna unguiculata*	Feijão bongolon	São Domingos	300
CV03Vu	*Vigna unguiculata*	Feijão bongolon	Assomada	560

Table S1. Eigenvalues, proportion of variability and mineral traits that contributed to the first four PCs (PCA) concerning the five most cultivated and traded food legume species in Cabo Verde.

**Components**	PC1	PC2	PC3	PC4
**Eigenvalues**	2.936	2.076	1.658	1.070
**% Variance explained**	29.36	20.76	16.58	10.70
**% Cumulative variance**	29.36	50.12	66.70	77.40
**Coefficients of variance**				
**B**	-0.503	0.098	-0.120	-0.201
**Ca**	-0.242	-0.559	0.091	-0.134
**Cu**	0.072	-0.099	-0.545	-0.216
**Fe**	0.059	-0.239	0.447	0.607
**K**	-0.396	-0.132	-0.237	0.158
**Mg**	-0.266	0.394	0.400	-0.020
**Mn**	0.347	0.485	0.112	-0.056
**P**	-0.420	0.215	0.009	0.147
**S**	-0.394	0.222	0.132	-0.485
**Zn**	-0.008	0.328	-0.484	0.492

Table S2. Accessions in worldwide genebanks of food legume species of Cabo Verde assessed through the Genesys Database [71].

Species	Number of accessions	Biological status of accessions	Provenance (Island)
Cajanus cajan	9	Breeding/Research Material	Unknown
	5	Traditional cultivar/Landrace	Unknown
Phaseolus lunatus	1	Traditional cultivar/Landrace	Unknown
Phaseolus vulgaris	1	Traditional cultivar/Landrace	Unknown
Vigna unguiculata	1	Traditional cultivar/Landrace	Santiago
